# Comparison of Methods to Identify Pathogens and Associated Virulence Functional Genes in Biosolids from Two Different Wastewater Treatment Facilities in Canada

**DOI:** 10.1371/journal.pone.0153554

**Published:** 2016-04-18

**Authors:** Etienne Yergeau, Luke Masson, Miria Elias, Shurong Xiang, Ewa Madey, Hongsheng Huang, Brian Brooks, Lee A. Beaudette

**Affiliations:** 1 National Research Council Canada, Energy Mining and Environment, Montreal, Qc, Canada; 2 National Research Council Canada, Human Health Therapeutics, Montreal, Qc, Canada; 3 Environment Canada, Biological Assessment and Standardization Section, Ottawa, On, Canada; 4 Canadian Food Inspection Agency, Fertilizer Safety Office, Plant Health & Biosecurity Directorate, Ottawa, On, Canada; 5 Canadian Food Inspection Agency, Ottawa Laboratory – Fallowfield, Ottawa, On, Canada; Wageningen University and Research Centre, NETHERLANDS

## Abstract

The use of treated municipal wastewater residues (biosolids) as fertilizers is an attractive, inexpensive option for growers and farmers. Various regulatory bodies typically employ indicator organisms (fecal coliforms, *E*. *coli* and *Salmonella*) to assess the adequacy and efficiency of the wastewater treatment process in reducing pathogen loads in the final product. Molecular detection approaches can offer some advantages over culture-based methods as they can simultaneously detect a wider microbial species range, including non-cultivable microorganisms. However, they cannot directly assess the viability of the pathogens. Here, we used bacterial enumeration methods together with molecular methods including qPCR, 16S rRNA and *cpn60* gene amplicon sequencing and shotgun metagenomic sequencing to compare pre- and post-treatment biosolids from two Canadian wastewater treatment plants (WWTPs). Our results show that an anaerobic digestion WWTP was unsuccessful at reducing the live indicator organism load (coliforms, generic *E*. *coli* and *Salmonella*) below acceptable regulatory criteria, while biosolids from a dewatering/pelletization WWTP met these criteria. DNA from other pathogens was detected by the molecular methods, but these species were considered less abundant. *Clostridium* DNA increased significantly following anaerobic digestion treatments. In addition to pathogen DNA, genes related to virulence and antibiotic resistance were identified in treated biosolids. Shotgun metagenomics revealed the widest range of pathogen DNA and, among the approaches used here, was the only approach that could access functional gene information in treated biosolids. Overall, our results highlight the potential usefulness of amplicon sequencing and shotgun metagenomics as complementary screening methods that could be used in parallel with culture-based methods, although more detailed comparisons across a wider range of sites would be needed.

## Introduction

Large wastewater treatment facilities start with clarification and end with disinfection of the liquid portion before discharging it into a nearby watercourse. The remaining non-liquid portion, sewage sludge, can undergo different biological as well as physical-chemical treatment processes by means of anaerobic or aerobic digestion, dewatering or pelletization [1]. Municipal biosolids, as defined by the Canadian Council of Ministers of the Environment (CCME), are organic-based products which may be solid, semi-solid or liquid and which are produced from the treatment of municipal sludge. Municipal biosolids are municipal sludge which has been treated to meet to jurisdictional standards, requirements or guidelines including the reduction of pathogens. It is estimated that 0.4 to 8 million tons of municipal biosolids are produced annually in Canada, USA and Europe [2–4]. A substantial amount of these biosolids are formulated into fertilizer for land application as a means of waste management [4, 5].

The recycling of organic wastes for land application as fertilizers and supplements (e.g., soil amendments) can result in benefits through the suppression of plant diseases [6], return and cycling of nutrients to the soil [7], and improvement of the physical properties of the soil (e.g. moisture absorbance) by increasing the overall organic matter content [8]. In contrast, there may also be risks associated with adding biosolids to soil, since these materials can be a potential source of pathogens, endotoxins and chemicals from industrial and household sources, which could lead to adverse environmental and human health effects [9–11]. As such, the benefits must be carefully balanced against the potential safety hazards associated with these materials. Consideration of the sources of waste-derived materials and the level of processing and treatment used during their manufacture are essential in determining the risks, since concerns over plant, animal, and human pathogens can be effectively alleviated with adequate treatment. Although very little is known of public health issues directly linked to pathogens in biosolids [10, 12, 13], direct contact or contamination of food crops represent two plausible routes whereby pathogens, if present in significant amounts, could affect human health. Pathogens of concern that may be present in sewage include: bacteria (e.g. *Salmonella* spp, *Escherichia coli* pathogenic strains, *Campylobacter jejuni*), viruses (e.g. Adenovirus, Rotavirus, Hepatitis A), protozoa (e.g. *Cryptosporidium* sp., *Entamoeba histolytica*, *Giardia lamblia*), and helminths (e.g. *Ascaris lumbricoides*, *Ascaris suum*, *Trichuris trichiura*) [14–16].

Pathogen inactivation is a key goal in biosolids production. Previous studies have shown that pathogens that have survived sewage treatment processes end up in biosolid-amended soils [9, 11]. Additionally, protozoan parasites including *Cryptosporidium* sp. and *Giardia* sp. were also reported to survive wastewater treatment processes [17]. Various regulatory bodies both domestically (at the provincial, territorial and federal level) as well as internationally, typically employ indicator organisms (fecal coliforms, *E*. *coli* and *Salmonella*) to assess the adequacy and efficiency of the treatment process in reducing pathogen loads in the final product. For example, according to the federal Canadian Food Inspection Agency (CFIA) *Salmonella* must be absent (non-detectable) and fecal coliform levels must not exceed 1000 MPN/g of dry weight in biosolids that are sold or imported into Canada [18]. These regulated levels vary between Canadian provinces and other countries and sometimes depend on the intended use of biosolids as fertilizers (e.g., food vs. non-food crops). These microbial indicators do not represent a comprehensive list of pathogens found in biosolids, but are used as indicators of treatment efficiency regarding pathogen inactivation. Since culture-based methods are used to enumerate these bacteria, they fail to provide information on non-indicator pathogens as well as viable but non-culturable (VBNC) organisms. Although qPCR-based quantification could circumvent some of these limitations, the large list of potential pathogens would render it a highly laborious and costly process. As such, a more holistic approach is needed to better characterize the pathogen population/load in treated biosolids intended for field application. In the present study, our goals were two-fold: 1) observe the effectiveness of different wastewater treatment processes through changes in the microbial taxonomical and functional community composition in the end product by a genomic approach and 2) compare traditional pathogen detection methods to modern molecular detection methods. To achieve the latter, bacterial enumeration (most probable number, MPN) methods were compared to indirect molecular detection techniques including qPCR, 16S rRNA and *cpn60* gene amplicon pyrosequencing and shotgun metagenomic sequencing in their ability to detect pathogens and virulence genes in biosolids obtained from two different WWTP. The WWTP used different treatments, namely anaerobic digestion and dewatering-pelletization, and samples were taken before and after treatment at various time points over the course of one year.

## Material and Methods

### Study sites and sample collection and characterization

Two Canadian biosolid treatment facilities named A and C by the authors were sampled at three time intervals in one year. The owners of the sites gave their consent to carry out this study on these sites. The Plant A treats waste activated sludge by anaerobic digestion and dewatering process with end product of wet pellets. Plant C treats waste activated sludge in a dewatering/pelletization process involving belt-filter press and a final process of pelletization by thermal drier (250–450°C at the entry, and 80–130°C at the exit). Samples were taken prior to and just after treatment in triplicate resulting in a total of 36 samples. The samples were transported on ice and were stored at 4°C (culture methods) or -20°C (DNA extraction) immediately after receiving until further use. The biosolid samples were labeled using the facility letter (A or C), treatment, and sampling date (Table 1). The moisture content (moisture %) of the samples was determined using an electronic moisture analyzer (IR-35 Moisture Analyzer, Denver Instrument Co, Bohemia, NY, USA). Culture-based methods and qPCR were performed on all samples while other molecular methods were performed on a subset of samples.

**Table 1:** Physical characteristics of pre- and post-treatment biosolids in two Canadian facilities. Values are mean ± SD.

Facility/ Operation	Treatment Method	Sampling date	Pre- or Post- Treatment	Sample appearance	Moisture content (%)	DNA content (μg/g of biosolids DW)
Plant A	Anaerobic digestion	Mar. 2009	Pre	Semi-solid	95.2±0.08	33.2±9.5
		Mar. 2009	Post	Wet pellets	71.9±1.27	390.5±28.2
		Aug. 2009	Pre	Semi-solid	94.7±0.46	237.6±74.2
		Aug. 2009	Post	Wet pellets	69.6±0.29	322.6±10.7
		Feb. 2010	Pre	Semi-solid	95.5±0.34	631.8±117.0
		Feb. 2010	Post	Wet pellets	73.4±0.66	60.3±27.5
Plant C	Dewatering/ pelletization	May 2009	Pre	Semi-solid	63.2±4.34	53.8±4.1
		May 2009	Post	Dry pellets	13.5±2.21	9.1±1.3
		Nov. 2009	Pre	Semi-solid	82.0±1.00	381.7±103.7
		Nov. 2009	Post	Dry pellets	11.4±0.29	2.7±0.8
		Mar. 2010	Pre	Semi-solid	97.2±0.14	232.6±71.5
		Mar. 2010	Post	Dry pellets	10.1±0.14	0.55±0.03

### Culture-based methods

Fecal coliforms and *E*. *coli* in each sample were evaluated using a most probable number assay (MPN) according to method MFHPB-19 [19]. Briefly, 90 ml of peptone water was added to 10g of sample (dry pellets were crushed before the addition of peptone water) followed by homogenization. Ten-fold serial dilutions of the suspension were made using peptone water. One ml aliquots of each dilution were inoculated into five tubes of lauryl sulfate tryptose (LST) broth. The production of gas in LST broth indicated a presumptive positive test for coliforms. The presence of coliforms was confirmed by the detection of gas after inoculation of positive LST broth cultures into brilliant green lactose bile (BGLB) broth, and incubation for 48 ± 4 h at 35°C. The presence of fecal coliforms was determined by the detection of gas production following inoculation of positive LST broth cultures into *E*. *coli* (EC) broth and incubation for 48 h at 45°C. To determine the presence of *E*. *coli*, the positive EC broth cultures were subcultured onto Levine's eosin methylene blue agar and the plates incubated for 18–24 h at 35°C. Colonies with a typical *E*. *coli* morphology were subcultured onto MacConkey agar. The isolates were presumptively identified as *E*. *coli* using preliminary biochemical tests [19] and confirmed using commercial biochemical test kits (Remel Micro ID, Oxoid and API-20E, bioMérieux Canada, Inc.). *Enterobacter cloacae* American Type Culture Collection (ATCC) 1307 and *E*. *coli* ATCC 11775 were included as positive controls and *Salmonella* Berta ATCC 8392 was included as a negative control. The probable level of the target organisms based on dry weight was then statistically calculated from a MPN table.

Quantitative detection of *Salmonella* was carried out based on method MFLP-75 [20]. The original qualitative method was modified to be used as an MPN procedure. Fifty ml of nutrient broth (NB) was added to 25 g of sample and incubated for 1 h at 35°C followed by addition of 175 ml of NB. Ten-fold serial dilutions of the homogenates were made using NB and the tubes (5 for each dilution) were incubated for 18–24 h at 35°C. A portion of each broth culture was inoculated onto Modified Semi-solid Rappaport Vassiliadis (MSRV) agar, and the plates incubated for 72 hours at 42°C. Presumptive *Salmonella* positive MSRV cultures were subcultured onto MacConkey agar. The selected colonies from MacConkey agar were initially tested for *Salmonella* using confirmatory test media including the agar slants of triple sugar iron (TSI) and lysine iron agar (LIA) and urea. The suspect isolates were purified using xylose lysine tergitol-4 (XLT-4) or xylose lysine deoxycholate (XLD) agar, and confirmed using biochemical (API 20E) and serological tests including agglutination (Oxoid *Salmonella* latex test, Oxoid) and an enzyme linked immune-sorbent assay (ELISA) [21]. *Salmonella* Typhimurium ATCC 14028 or *S*. Berta ATCC 8392 was used as a positive control, and *E*. *coli* ATCC 11775 or *Enterobacter cloacae* ATCC 1307 used as a negative control. The MPN of *Salmonella* per g of dry sample was calculated based on dry weight.

### DNA extraction

DNA was extracted using the PowerMax^®^ Soil DNA Isolation Kit following the manufacturer’s instructions (MoBio, Carlsbad, CA). The final purified DNA was then used for PCR amplification or stored at -20°C.

### 16S rRNA and *cpn60* gene sequencing

In order to taxonomically identify microbes potentially present in biosolids and observe shifts in community composition, amplicon sequencing of two distinct marker genes was carried out. For the 16S rRNA gene, eight libraries were prepared using two different universal bacterial primer sets on four different samples (“Plant A August 2009 before-after” and “Plant C May 2009 before-after”). The following primers were used: V1–V3: forward 5’ CGTATCGCCTCCCTCGCGCCATCAG*ACGAGTGCGT***AGTTTGATCCTGGCTCAG-3’**, reverse 5’- CTATGCGCCTTGCCAGCCCGCTCAG*ACGCTCGACA***CATTACCGCGGCTGCTGG-3’** and V3-5: forward 5’- CGTATCGCCTCCCTCGCGCCATCAG*AGACGCACTC***GCCTACGGGAGGCAGCAG-3’** reverse 5’- CTATGCGCCTTGCCAGCCCGCTCAG*AGCACTGTAG***CCGTCAATTCMTTTRAGT-3’** where the italic sequence represents the sample specific multiplex identifier, the bold sequence represents the template specific sequences and the remaining sequence is the 454 adapter A (forward) and adapter B (reverse).

For the *cpn60* gene, four libraries were prepared using the “Plant A August 2009 before-after” and “Plant C May 2009 before-after” samples. The following universal primers were used: for “Plant A August 2009 before” and “Plant C May 2009 before”, forward: 5’- CGTATCGCCTCCCTCGCGCCATCAG*ATCAGACACG***GCIGGIGAYGGNACNACNAC3’**, reverse: 5’- CTATGCGCCTTGCCAGCCCGCTCAG*ATATCGCGAG***TCICCRAANCCNGGNGCYTT-‘3**. The same primers were used for “Plant A August 2009 after” and “Plant C May 2009 after” except that the multiplex identifiers (in italics) were replaced by the following: forward primer: CGTGTCTCTA and reverse primer: CTCGCGTGTC. The PCR conditions were as follows: the mixture in a 50ul final volume contained 50ng DNA, 25pmol of each primer for 16S or *cpn60*, 1X final Taq polymerase buffer, and 2.5 units of Taq polymerase (New England BioLabs Ltd, Pickering, ON, Canada) and 1 μl of 10 mM deoxynucleoside triphosphates. PCR cycling consisted of 94°C for 5 minutes, denaturation at 94°C for 30 seconds, annealing at 56°C for 30 seconds for 16S V3-1, 50°C for 16S V5-3 and 55°C for *cpn60*, with an extension at 72°C for 45 seconds after 35 cycles. A final extension at 72°C was added for 7 minutes.

### Real-time quantitative PCR

The abundance of *E*. *coli* was quantified using two distinct sets of primers targeting the beta-D-glucuronidase gene (*uidA*): uidA1-F: CAGCAATTGCCCGGCTTTCTTGTA, uidA1-R: GGCATTCAGTCTGGATCGCGAAA (generating a fragment of 83bp) and uidA2-F: GTATCGGTGTGAGCGTCGCAG and uidA2-R: GCGTGGTGATGTGGAGTATTGCC (generating a fragment of 154 bp). For *Salmonella* quantification, two sets of primers targeting the invasion gene A (*invA* and *sal*) were used: invA-F: GATTCTGGTACTAATGGTGATGATC, invA-R: GCCAGGCTATCGCCAATAAC (generating a fragment of 287 bp) and sal-F: GCGTTCTGAACCTTTGGTAATAA, and sal-R: CGTTCGGGCAATTCGTTA (generating a fragment of 102bp) [22]. Real-time quantitative PCR (qPCR) amplification was performed using a Rotor Gene 3000 instrument (Corbett Research, Mortlake, NSW, Australia) using a QuantiTect SYBR Green PCR master mix (Qiagen) in a 20 μl volume containing 10 pmol of each primer and a final MgCl_2_ concentration of 2.5mM for *uidA1* and 3.5mM for *uidA2*, *invA* and *sal*. The amplification conditions were as follows: 95°C for 15 minutes, followed by 40 cycles of 95°C for 10 seconds, 55°C for 15 seconds and 72°C for 15 seconds. Fluorescence was measured at the end of each cycle at 72°C and a melting curve analysis (65–95°C) was performed at the end of the amplification procedure.

Standard curves were generated from PCR fragments using the above mentioned primer sets and genomic DNA from *E*. *coli* K12 and *Salmonella enterica* serovar Typhimurium. Amplicons from the different primer sets were cloned using the pGEM-T easy Vector System (Promega U.S. Madison, WI) and transformed into *E*. *coli* (JM109). Recombinant plasmids were isolated and linearized with *Sca*I (New England BioLabs) quantified by PicoGreen (Invitrogen) and used to generate standard curves with serial dilutions. Standard curve efficiencies were all between 0.95 and 1.0 with R^2^ value between 0.99 and 1.00.

### Amplicon and shotgun metagenomic sequencing

Roche 454 GS FLX Titanium sequencing (454 Life Sciences, Branford, CT, USA) was performed at McGill University and Genome Quebec Innovation Center, Montreal, QC, Canada. Amplicons and metagenomic samples were sequenced on a single run using an eight lane gasket. The 16S amplicons from “Plant A August 2009 before”, “Plant A August 2009 after”, “Plant C May 2009 before”, “Plant C May 2009 after” used one lane each (the two different primer sets V3-1 and V5-3 of the same sample were multiplexed in the same lane). The *cpn60* amplicons from “Plant A August 2009” and the “Plant C May 2009” used two individual lanes (before and after samples were multiplexed in the same lane). The shotgun metagenomic samples (“Plant A August 2009 before” and “Plant A August 2009 after”) used one lane each.

### Sequence data analysis

16S sequence data were primarily analyzed through the RDP pyrosequencing pipeline (http://pyro.cme.msu.edu/). The sequences were deconvoluted and binned according to their multiplex identifier (only accepting perfect matches), and the multiplex identifier and the forward primer were trimmed using the ‘Pipeline Initial Process’ tool. This resulted in eight distinct data sets. Using the ‘Pipeline Initial Process’ tool, all sequences that contained undetermined bases (N) or were shorter than 150 bp were removed. The data sets were submitted to the RDP Classifier tool using a bootstrap cutoff of 80%. The datasets from the two different 16S primers were then compared and since no large variation were observed (data not shown), the datasets were pooled for all downstream analyses. The *cpn60* sequences were subjected to the same initial process using the tools available in the RDP pyrosequencing pipeline. The four sequence datasets were then compared to the cpnDB (http://cpndb.cbr.nrc.ca/) using blastn [23]. Blast results were analyzed using MEGAN to place the sequences in the NCBI taxonomy using a lowest common ancestor algorithm [24].

For shotgun metagenomic datasets, replicate sequences that resulted from the attachment of DNA to beads during emulsion PCR and were not derived independently from the environmental data were removed from the dataset using the method of Gomez-Alvarez [25]. Sequences were then submitted to MG-RAST v. 2.0 for automated annotation [26]. The abundance of different taxa given by MG-RAST was further normalized to the individual genome size by dividing the number of hits by the individual genome sizes. This normalization is necessary due to variability in genome size between different organisms as larger genomes generate more reads even though the organism is not more abundant in the sample. Data mining efforts were specifically focused on known pathogens and virulence genes. Results are presented as the raw number of sequences related to a particular function or taxon, or the relative abundance of taxa calculated from the number of reads mapping to this taxon divided by the total number of reads in the dataset.

### Statistical analyses

Two proportion z-tests were performed according to Wang [27]. Principal coordinate analysis (PCoA) were performed based on Bray-Curtis distance calculated from genus relative abundance in R [28] using the vegan package [29]. Spearman rank-order correlations were performed in R.

## Results

### Physical characteristics of biosolid samples

A total of 36 samples were taken at three different dates from two wastewater treatment plants. The two different plants used different methods to treat the biosolids: Plant A used anaerobic digestion, which lowered the water content in the biosolids from 95% to approximately 70%, while Plant C used a dewatering/pelletization treatment which reduced the biosolids water content from 63–97% to approximately 10% (Table 1).

### Taxonomical shifts following biosolids treatment

**Fig 1:**
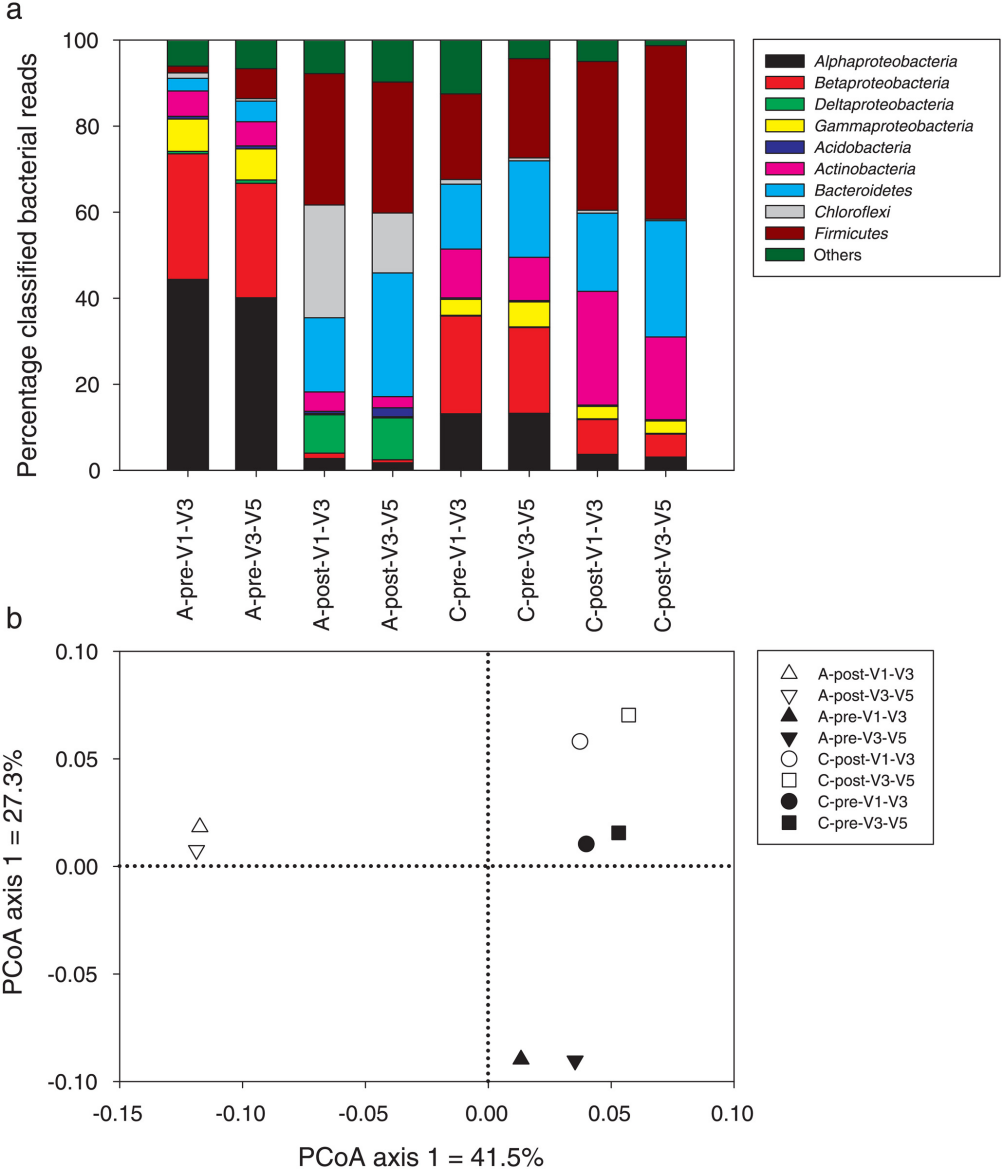
Bacterial phylum-level community composition (a) and genus-level principal coordinate analysis (PCoA) ordination (b) for 16S rRNA gene sequencing of the V1–V3 and V3–V5 regions for pre- and post-treatment biosolid sampled at Plant A on August 4, 2009 and Plant C on May 12, 2009. Solid symbols: pre-treatment, empty symbols: post-treatment.

**Fig 2:**
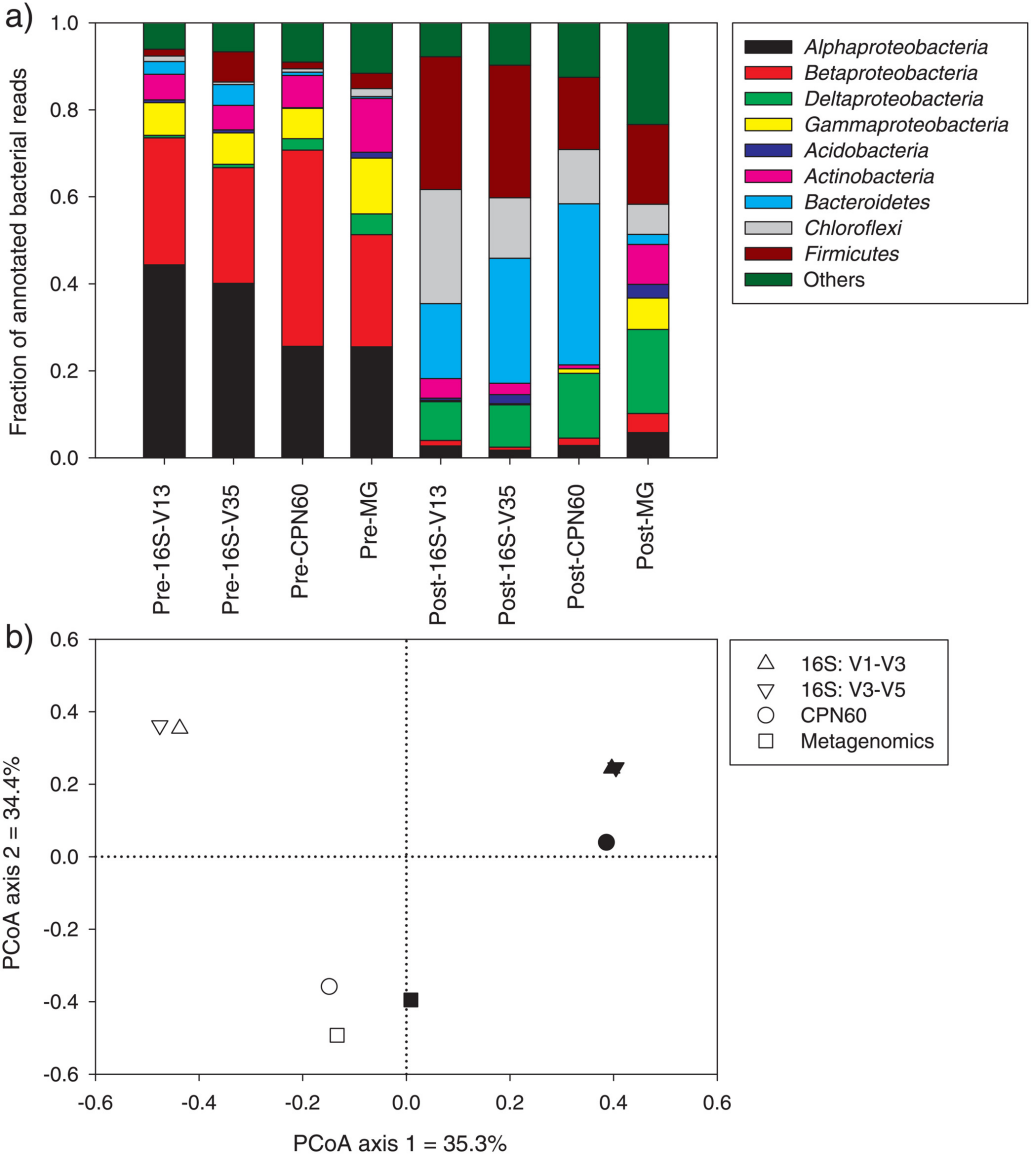
Bacterial phylum-level community composition (a) and genus-level principal coordinate analysis (PCoA) ordination (b) for 16S rRNA gene sequencing of the V1–V3 and V3–V5 regions, *cpn60* gene sequencing and shotgun metagenomic sequencing for pre- and post-treatment biosolid sampled at Plant A on August 4, 2009. Solid symbols: pre-treatment, empty symbols: post-treatment.

One pre/post sample pair from Plant A (August 4, 2009) and another pair from Plant C (May 12, 2009) were selected for amplicon sequencing (16S and *cpn60*). For both plants, large changes were observed at the phylum/class level following treatment independent of the primer pair used (Fig 1a). For Plant A, *Proteobacteria* dominated the microbial community before treatment and was replaced by *Firmicutes*, *Bacteroidetes* and *Chloroflexi* following treatment (Fig 1a). For Plant C, the shifts were less drastic, with decreases in the relative abundance of *Proteobacteria* and increases in *Actinobacteria*, *Bacteroidetes*, and *Firmicutes* following treatment (Fig 1a). When looking at lower taxonomical levels using Unifrac analyses, a similar pattern emerged with the anaerobic digestion from Plant A causing stronger shifts in microbial communities (dots more distant) and the dewatering/pelletization from Plant C causing less dramatic shifts (Fig 1b). Similar results were obtained from *cpn60* amplicon analyses for Plant A (Fig 2) and Plant C (not shown). Shifts in the dominant genera were also observed for the two plants following treatments using 16S rRNA and *cpn60* gene sequencing. For Plant A, the community shifted from an *Acidovorax* and *Novosphingobium* dominated community (Table 2) to one dominated by anaerobes and syntrophic bacteria like *Syntrophus*, *Sedimentibacter*, *Prevotella*, *Clostridium* and *Thermovirga* (Table 3). For Plant C, the shifts were less evident, with a community that was generally dominated by *Paludibacter* and *Microbacterium* both before and after the biosolid treatment (Table 4).

**Table 2:** Comparison of the 20 most abundant genera determined by different sequencing methods in Plant A biosolids, pre-treatment (August 4, 2009).

16S-V1-3		16S-V3-5		*cpn60*		Metagenomics	
Genus	RA	Genus	RA	Genus	RA	Genus	Rel. Abund.
*Acidovorax*	22.6	*Novosphingobium*	22.1	*Acidovorax*	19.4	*Mycobacterium*	4.2
*Novosphingobium*	17.7	*Acidovorax*	16.0	*Rhodobacter*	17.0	*Acidovorax*	3.9
*Thermomonas*	8.0	*Faecalibacterium*	11.6	*Novosphingobium*	10.1	*Erythrobacter*	3.5
*Pseudorhodobacter*	5.6	*Rhodobacter*	9.4	*Leptothrix*	5.6	*Pseudomonas*	3.0
*Hydrogenophaga*	4.3	*Thermomonas*	2.6	*Variovorax*	4.9	*Moraxella*	1.9
*Phenylobacterium*	3.5	*Phenylobacterium*	2.2	*Ancylobacter*	3.8	*Sphingopyxis*	1.8
*Rhodobacter*	2.9	*Pseudorhodobacter*	2.2	*Rhodoferax*	2.8	*Rubrivivax*	1.8
*Simplicispira*	2.8	*Devosia*	2.2	*Janibacter*	2.2	*Rhodoferax*	1.7
*Roseomonas*	1.9	*Propionicicella*	1.6	*Pirellula*	1.9	*Polaromonas*	1.6
*Devosia*	1.9	*Hydrogenophaga*	1.4	*Plesiocystis*	1.9	*Propionibacterium*	1.6
TM7 genus	1.7	*Geothrix*	1.4	*Akkermansia*	1.4	*Caulobacter*	1.5
*Caenimonas*	1.4	*Curvibacter*	1.4	*Mycobacterium*	1.4	marine actinobacterium PHSC20C1	1.4
*Altererythrobacter*	1.4	*Simplicispira*	1.2	*Anaeromyxobacter*	1.4	*Sphingomonas*	1.4
*Tetrasphaera*	1.4	*Aquabacterium*	1.0	*Chthoniobacter*	1.3	*Xanthomonas*	1.3
*Geothrix*	1.4	*Roseomonas*	1.0	*Curvibacter*	1.3	*Verminephrobacter*	1.3
*Zoogloea*	1.3	*Rhodoferax*	0.9	*Sphingomonas*	1.3	*Delftia*	1.3
*Sphingomonas*	0.9	*Microbacterium*	0.8	*Azoarcus*	1.2	*Flavobacterium*	1.3
*Caulobacter*	0.9	*Trichococcus*	0.7	*Deinococcus*	1.1	*Rhodobacter*	1.3
*Thauera*	0.7	*Caulobacter*	0.6	*Agreia*	1.1	*Janibacter*	1.2

RA = relative abundance which is presented as the percentage of all reads classified at the genus level.

**Table 3:** Comparison of the 20 most abundant genera determined by different sequencing methods in Plant A biosolids, post-treatment (August 4, 2009).

16S-V1-3		16S-V3-5		*cpn60*		Metagenomics	
Genus	RA	Genus	RA	Genus	RA	Genus	Rel. Abund.
*Sedimentibacter*	22.7	*Sedimentibacter*	33.0	*Syntrophus*	14.2	*Syntrophus*	5.1
*Syntrophorhabdus*	16.6	*Syntrophorhabdus*	17.9	*Prevotella*	12.7	*Mycobacterium*	4.6
*Thermovirga*	15.6	*Thermovirga*	15.6	*Heliobacterium*	5.9	*Clostridium*	4.0
*Smithella*	12.5	*Petrimonas*	6.8	*Bacteroides*	5.3	*Streptococcus*	3.3
*Anaerovorax*	2.9	Gp10 genus	5.0	*Cloacamonas*	4.5	*Bacteroides*	2.5
OP10 genus	2.8	*Smithella*	4.2	*Thermanaerovibrio*	4.5	*Geobacter*	2.3
*Clostridium*	2.7	*Levilinea*	1.9	*Clostridium*	3.2	*Thermoanaerobacter*	2.3
*Turicibacter*	1.4	*Sporacetigenium*	1.2	*Thermomicrobium*	2.6	*Dehalococcoides*	2.2
*Acidovorax*	1.2	*Syntrophomonas*	0.8	*Desulfobacterium*	2.5	*Bacillus*	2.1
*Petrimonas*	1.2	*Ruminococcus*	0.6	*Herpetosiphon*	2.4	*Propionibacterium*	1.7
*Cryptanaerobacter*	1.1	*Anaerovorax*	0.6	*Oxobacter*	2.3	*Roseiflexus*	1.7
*Syntrophomonas*	0.9	*Clostridium*	0.6	*Geobacter*	2.1	*Parabacteroides*	1.6
*Ruminococcus*	0.9	*Turicibacter*	0.5	*Rhodospirillum*	2.1	*Syntrophobacter*	1.5
*Microbacterium*	0.8	*Gracilibacter*	0.5	*Thermosinus*	2.0	*Thermotoga*	1.5
*Mycobacterium*	0.8	*Cloacibacillus*	0.5	*Desulforudis*	1.8	*Salmonella*	1.5
*Trichococcus*	0.8	*Propionicicella*	0.5	*Chlorobium*	1.7	*Desulfococcus*	1.4
*Chelatococcus*	0.7	*Pelotomaculum*	0.4	*Sphaerobacter*	1.7	*Moorella*	1.4
*Caldilinea*	0.6	*Chelatococcus*	0.4	*Desulfovibrio*	1.5	*Desulfovibrio*	1.4
*Bellilinea*	0.6	*Trichococcus*	0.4	*Alistipes*	1.4	*Alkaliphilus*	1.4

RA = relative abundance which is presented as the percentage of all reads classified at the genus level.

**Table 4:** Comparison of the 20 most abundant genera determined by 16S amplicon sequencing in Plant C biosolids (May 12, 2009).

Pre-treatment				Post-treatment			
Genus	V1–V3	Genus	V3–V5	Genus	V1–V3	Genus	V3–V5
TM7 genus	6.84	*Paludibacter*	6.33	*Microbacterium*	8.43	*Paludibacter*	10.54
*Paludibacter*	4.14	*Propionicicella*	2.16	*Paludibacter*	8.27	*Faecalibacterium*	6.84
*Microbacterium*	2.58	*Acidovorax*	1.95	*Clostridium*	5.16	*Sporacetigenium*	4.72
*Trichococcus*	2.09	*Flavobacterium*	1.74	TM7 genus	3.04	*Clostridium*	4.25
*Dechloromonas*	2.01	*Trichococcus*	1.50	*Sporacetigenium*	1.24	*Propionicicella*	3.89
*Acidovorax*	1.88	*Clostridium*	1.28	*Dechloromonas*	0.99	*Flavobacterium*	1.82
*Clostridium*	1.51	*Pseudomonas*	1.16	*Turicibacter*	0.98	*Microbacterium*	1.78
*Prevotella*	1.49	*Ferruginibacter*	1.07	*Flavobacterium*	0.93	*Ferruginibacter*	0.64
*Flavobacterium*	1.31	*Rhodobacter*	1.06	*Trichococcus*	0.81	*Trichococcus*	0.61
*Zoogloea*	1.18	*Nitrospira*	0.92	*Prevotella*	0.53	*Turicibacter*	0.49
*Janthinobacterium*	1.04	*Janthinobacterium*	0.90	*Tetrasphaera*	0.52	*Dechloromonas*	0.41
*Nitrospira*	1.00	*Microbacterium*	0.87	*Zoogloea*	0.51	*Rhodobacter*	0.30
*Ferruginibacter*	0.95	*Devosia*	0.73	*Janthinobacterium*	0.47	*Bacillus*	0.28
*Polaromonas*	0.66	*Zoogloea*	0.66	*Acetanaerobacterium*	0.41	*Acetivibrio*	0.28
*Pseudorhodoferax*	0.58	*Novosphingobium*	0.63	*Ferruginibacter*	0.38	*Acidovorax*	0.27
*Novosphingobium*	0.55	*Ferribacterium*	0.63	*Acidovorax*	0.27	*Bacteroides*	0.26
*Simplicispira*	0.47	*Polaromonas*	0.56	*Bacillus*	0.26	*Ferribacterium*	0.26
*Tetrasphaera*	0.34	*Faecalibacterium*	0.50	*Faecalibacterium*	0.25	*Zoogloea*	0.26
*Hyphomicrobium*	0.33	*Xylanibacter*	0.48	*Pseudorhodoferax*	0.17	*Janthinobacterium*	0.25
*Rhodoferax*	0.28	*Dechloromonas*	0.40	*Micropruina*	0.17	*Xylanibacter*	0.22

RA = relative abundance which is presented as the percentage of all reads classified at the genus level.

For potential pathogens, we also looked at the species level using the metagenomics datasets from Plant A. Anaerobic digestion was successful at reducing the relative abundance of several species, including *E*. *coli*, *Legionella* and *Pseudomonas* species (Table 5). For *E*. *coli*, this relative decrease was also seen for most samples examined by qPCR (*uidA1* shown in Table 6, with *uidA2* showing identical trends) and MPN (Table 6). MPN analyses also highlighted a general decrease in *Salmonella* abundance following treatments (Table 6), while *Salmonella* qPCR (*invA* and Sal) was below the qPCR detection limit for all samples. However, anaerobic digestion increased the relative abundance of *Campylobacter*, *Chlamydia*, *Clostridium*, *Enterococcus*, *Listeria* and *Staphylococcus* species DNA in the metagenomic datasets (Table 5).

**Table 5:** Number of hits in metagenomic datasets matching selected species/genera containing pathogenic strains in biosolids samples from Plant A sampled on August 4, 2009.

Genera/species	Pre-	Post-			Pre-	Post-	
More				Less			
*Bacillus*	1242	3684	***	*Aeromonas*	529	308	***
*B*. *anthracis*	54	164	***	*A*. *hydrophila*	289	183	**
*B*. *subtilis*	109	336	***	*A*. *salmonicida*	240	125	***
*B*. *thuringiensis*	74	174	***	*Agrobacterium*	1075	263	***
*Campylobacter*	137	288	***	*A*. *rhizogenes*	23	5	**
*C*. *fetus*	28	47	**	*A*. *tumefaciens*	1052	258	***
*C*. *jejuni*	27	59	***	*Brucella*	751	189	***
*C*. *coli*	24	31	NS	*B*. *abortus*	370	85	***
*Chlamydia*	17	57	***	*Burkholderia*	5337	1347	***
*Chlamydophila*	27	170	***	*B*. *cenocepacia*	1086	275	***
*C*. *pneumoniae*	6	73	***	*B*. *cepacia*	1215	315	***
*Clostridium*	2037	10026	***	*B*. *mallei*	150	39	***
*C*. *botulinum*	189	925	***	*B*. *pseudomallei*	687	210	***
*C*. *difficile*	204	870	***	*Corynebacterium*	796	487	***
*C*. *perfringens*	130	645	***	*C*. *diphtheriae*	135	69	***
*C*. *tetani*	120	743	***	*C*. *jeikeium*	112	96	NS
*Enterococcus*	141	436	***	*C*. *striatum*	3	6	NS
*E*. *faecalis*	93	268	***	*Escherichia coli*	420	260	***
*E*. *faecium*	48	168	***	*Flavobacterium*	4186	3134	***
*Helicobacter*	73	112	***	*F*. *johnsoniae*	2223	1564	***
*H*. *pylori*	32	22	NS	*F*. *psychrophilum*	1032	506	***
*Listeria*	209	466	***	*Klebsiella*	88	52	*
*L*. *monocytogenes*	131	390	***	*K*. *pneumoniae*	88	52	*
*Salmonella*	265	239	NS	*Legionella*	468	285	***
*S*. *bongori*	63	53	NS	*L*. *pneumophila*	468	285	***
*S*. *enterica enterica* serovar Dublin	24	26	NS	*Mycobacterium*	9100	7269	***
*S enterica enterica* serovar Paratyphi	27	14	NS	*M*. *avium*	946	708	*
*Staphylococcus*	144	377	***	*M*. *bovis*	219	187	NS
*S*. *aureus*	47	129	***	*M*. *leprae*	106	83	NS
*Streptococcus*	398	1060	***	*M*. *marinum*	1126	928	NS
*S*. *agalactiae*	33	101	***	*M*. *microti*	471	389	NS
*S*. *equi*	29	74	***	*M smegmatis*	1814	1523	NS
*S*. *pneumoniae*	44	114	***	*M*. *tuberculosis*	122	112	NS
*S*. *pyogenes*	68	181	***	*Pseudomonas*	4827	1590	***
*S*. *thermophilus*	26	118	***	*P*. *aeruginosa*	996	295	***
*Vibrio*	991	938	**	*P*. *fluorescens*	1156	386	***
*V*. *cholerae*	266	190	NS	*P*. *mendocina*	776	210	***
*V*. *parahaemolyticus*	55	62	NS	*Ralstonia*	4068	678	***
*V*. *vulnificus*	169	173	NS	*R*. *solanacearum*	1081	233	***
				*Shewanella*	1590	1219	*
				*S*. *putrefaciens*	111	55	**
				*Yersinia*	409	287	*
				*Y*. *enterocolitica*	63	64	NS

*P<0.05,

**0.05<P<0.01,

***P<0.001,

based on a two-proportion z-test.

**Table 6:** Mean (± SD) abundance of *E*. *coli*, coliforms and *Salmonella* based on qPCR (per g) and MPN counts (per 10 g).

Plant	Date	Pre-	Post-	% change
**qPCR**				
*uidA1*				
A	25/03/2009	0±0	2,681±1,333	NA
C	12/05/2009	50,183±13,154	30,713±3,793	-38.80
A	04/08/2009	2,059±966	4,769±5,266	131.61
C	23/11/2009	77,888±26,626	20,268±6,972	-73.98
A	08/02/2010	1,214±338	2,390±1,540	96.94
C	22/03/2010	1,714±404	4,937±1,245	188.11
**MPN**				
Coliform				
A	25/03/2009	10.9x10^6^±5.3x10^6^	2.8x10^6^±3.5x10^6^	-74.68
C	12/05/2009	3.7x10^8^±5.0x10^8^	0±0	-100.00
A	04/08/2009	1.9x10^8^±0.16x10^8^	2.8x10^7^±4.0 x10^7^	-85.04
C	23/11/2009	3.4x10^8^±1.0x10^8^	2±3	-100.00
A	08/02/2010	8,9x10^6^±5,2x10^6^	1.5x10^6^±1.0 x10^6^	-83.71
C	22/03/2010	9.9x10^5^±5.2x10^5^	0±0	-100.00
*E*.*coli*				
A	25/03/2009	5.5x10^5^±2.0x10^5^	1,304±1,588	-99.76
C	12/05/2009	3.2x10^8^±5.4X10^8^	0±0	-100.00
A	04/08/2009	9.2x10^6^±8.8x10^6^	7.6x10^6^±2.8x10^6^	-17.40
C	23/11/2009	6.9x10^7^±5.8x10^7^	0±0	-100.00
A	08/02/2010	8.6x10^6^±9.0x10^6^	1.5x10^6^±1.0x10^6^	-83.12
C	22/03/2010	72,787±29,338	0±0	-100.00
*Salmonella*				
A	25/03/2009	1±2	1±1	-37.48
C	12/05/2009	3±1	0±0	-100.00
A	04/08/2009	2,111±861	382±543	-81.90
C	23/11/2009	0±0	0±0	NA
A	08/02/2010	8,571±4,359	1.2x10^5^±1.1x10^5^	1307.91
C	22/03/2010	26±40	0±0	-100.00

NA: Not available (division by zero)

*uidA2* quantification showed trends identical to uidA1.

Salmonella quantification by qPCR (*invA* and *Sal*) was below detection limit for all samples.

### Functional shifts following biosolids treatment

Within the metagenomic datasets (Plant A, August 4, 2009), we focused our attention on functional genes related to pathogenicity and virulence (“Virulence” Subsystem hierarchy 1 category in MG-RAST). Although all subsystems were still detectable after the treatment, several of the “Virulence” subsystems decreased significantly, especially in the “Resistance to antibiotics and toxic compounds” Subsystem level 2 category (Table 7). In contrast, some subsystems were significantly more abundant following treatment. Some relatively abundant (more than 500 hits) subsystems like “Multidrug Resistance Efflux Pumps” and “Resistance to fluoroquinolones” were relatively more abundant in the biosolids following treatment (Table 7). The total relative abundance of “Virulence” related reads decreased following treatment, from 7.4% to 5.5% of total classified reads.

**Table 7:** Number of hits to related to virulence genes in the Plant A biosolids samples sampled on August 4, 2009.

Subsystem Hierarchy 2	Subsystem Name	Pre	Post	
**Less abundant following treatment**				
Adhesion	Widespread colonization island	280	135	***
Iron scavenging mechanisms	Hemin transport system	379	50	***
	Heme, hemin uptake and utilization systems in gram positive bacteria	116	54	***
	Pyoverdine biosynthesis new	188	108	***
Pathogenicity islands	*Listeria* Pathogenicity Island LIPI-1 extended	22	4	**
Prophage, transposon	Tn552	118	28	***
Resistance to antibiotics and toxic compounds	Cobalt-zinc-cadmium resistance	1692	876	***
	Multidrug resistance, tripartite systems found in gram negative bacteria	289	67	***
	Tolerance to colicin E2	122	24	***
	Acriflavin resistance cluster	914	567	***
	The mdtABCD multidrug resistance cluster	36	2	***
	MexE-MexF-OprN multidrug efflux system	28	1	***
	Mercury resistance operon	33	5	***
	Beta-lactamase	526	369	**
	Multiple antibiotic resistance MAR locus	14	3	*
	Methicillin resistance in Staphylococci	66	38	*
	Multidrug resistance, 2-protein version found in gram positive bacteria	38	19	*
Type III, Type IV, ESAT secretion systems	Type 4 secretion and conjugative transfer	1299	112	***
	Type III secretion system orphans	184	63	***
	Type 4 conjugative transfer system, IncI1 type	25	6	**
Type VI secretion systems	Type VI secretion systems	127	66	***
Unclassified	Ton and Tol transport systems	2421	1239	***
	Bacterial cyanide production and tolerance mechanisms	22	1	***
**More abundant following treatment**				
Adhesion	Adhesion of *Campylobacter*	18	47	***
	*Streptococcus pyogenes* recombinatorial zone	6	16	*
Invasion and intracellular resistance	*Listeria* surface proteins: LPXTG motif	2	11	**
	*Listeria* surface proteins: internalin-like proteins	168	184	*
Pathogenicity islands	Staphylococcal pathogenicity islands SaPI	92	140	***
Posttranslational modification	N-linked glycosylation in bacteria	97	235	***
	Pseudaminic acid biosynthesis	8	16	*
Prophage, transposon	Staphylococcal phi-Mu50B-like prophages	10	33	***
	Bacterial endolysins: autolysins, phage, and phage-like lysins	10	27	**
	IbrA and IbrB: co-activators of prophage gene expression	1	10	**
	*Listeria* phi-A118-like prophages	41	56	*
Resistance to antibiotics and toxic compounds	Multidrug resistance efflux pumps	1087	1337	***
	Resistance to fluoroquinolones	420	590	***
	Zinc resistance	125	208	***
	Resistance to vancomycin	16	33	**
	Tetracycline resistance, ribosome protection type	14	27	*
Toxins and superantigens	Streptolysin S biosynthesis and transport	6	24	***
Unclassified	*Streptococcus pyogenes* virulome	6	26	***

*P<0.05,

**0.05<P<0.01,

***P<0.001,

based on a two-proportion z-test.

### Method comparison

The detection of selected pathogens was compared for the different methods used for the samples taken on August 4, 2009 in Plant A (Table 8). *E*. *coli* was detected using MPN, qPCR and metagenomics sequencing, but not by amplicon sequencing, while *Salmonella* was detected by MPN and metagenomic sequencing (Table 8). Most of the other genera containing potential pathogens were only detected by metagenomic sequencing, with some exceptions like *Clostridium* that was also detected by amplicon sequencing in pre- and post-treatment samples (Table 8).

**Table 8:** Detection of selected selected species/genera containing pathogenic strains in biosolids from Plant A on August 4, 2009.

Pathogen	MPN	qPCR	16S V13	16S V5	*cpn60*	Metagenomics
	CFU/g biosolid dw	gene copies /g biosolid dw	Nb reads	Nb reads	Nb reads	Nb reads
Pre-						
*E*. *coli*	9.17x10^5^	2060 (*uidA1*) 2600 (*uidA2*)	0	0	0	420
*Salmonella*	211	ND	0	0	0	265
*Chlamydia*	-	-	0	0	0	17
*Chlamydophila*	-	-	0	0	0	27
*Clostridium*	-	-	11	6	0	2037
*Campylobacter*	-	-	0	0	0	137
*Enterococcus*	-	-	1	0	0	141
*Listeria*	-	-	0	0	0	209
*Staphylococcus*	-	-	0	0	0	144
*Legionella*	-	-	1	0	0	468
*Klebsiella*	-	-	0	2	0	88
*Yersinia*	-	-	0	0	0	409
Post-						
*E*. *coli*	7.58x10^5^	4769 (*uidA1*) 12493 (*uidA2*)	0	0	0	260
*Salmonella*	1000	ND	0	0	15	269
*Chlamydia*	-	-	0	0	0	57
*Chlamydophila*	-	-	0	0	5	170
*Clostridium*	-	-	134	26	1850	10026
*Campylobacter*	-	-	0	0	190	288
*Enterococcus*	-	-	0	1	0	436
*Listeria*	-	-	0	0	0	466
*Staphylococcus*	-	-	0	0	0	377
*Legionella*	-	-	0	0	20	285
*Klebsiella*	-	-	0	0	0	52
*Yersinia*	-	-	0	0	0	287

16S: average of 22517 sequences, 14221–32204.

*cpn60*: 4765 (pre) and 73466 (post) reads.

Metagenomes: 491,709 (pre) and 512,552 (post) reads.

ND: not detected, below qPCR detection limit.

All quantification methods revealed low abundance of coliforms, *E*. *coli* and *Salmonella*, being often below the detection limit, especially in the case of *Salmonella* (Table 6). The quantification of *E*. *coli* by MPN and qPCR methods was compared by Spearman correlation analysis, while the tests were not performed for *Salmonella* as the qPCR results were below detection limits in all cases. The abundance of *E*. *coli* measured by qPCR and MPN were not significantly correlated (uidA1 vs. MPN: r_s_ = 0.165, P = 0.261; uidA2 vs. MPN: r_s_ = 0.218, P = 0.137). The two qPCR quantification methods used (*uidA1* and *uidA2*) were significantly correlated to each other (r_s_ = 0.839, P<0.0001).

The community composition patterns were compared between the datasets from metagenomic and amplicon sequencing (two 16S primer pairs and one *cpn60* primer pair). The four different methods gave relatively similar patterns at the phylum/class level, especially when comparing the pre-treatment vs. the post-treatment samples (Fig 2a). When looking at the genus relative abundance using principal coordinate analysis (PCoA) of Bray-Curtis distances, the main difference observed was between pre- and post-treatment samples, with pre- and post-treatment samples being separated on the first axis of the ordination for both amplicon (16S and *cpn60*) and metagenomic datasets (Fig 2b). The samples analysed using metagenomic sequencing were less differentiated than the other samples (Fig 2b). For the 16S rRNA gene amplicon sequencing, both primer pairs clustered tightly together (Fig 2b). Similarly, when also including samples from Plant C, using different primer pairs resulted in very little difference (Fig 1b). When comparing the most abundant genera, the different sequencing methods generally resulted in similar results, even though the order and relative abundance of the dominant genera changed (Tables 2, 3 and 4). In most cases, the metagenomic analysis resulted in the largest differences (Tables 2 and 3). One interesting difference is that the metagenomic analysis resulted in a more even distribution of the different genera, with relative abundances never exceeding 5.1% as compared to 33% for amplicon sequencing (Tables 2 and 3).

## Discussion

### Pathogens and virulence genes in treated biosolids

After treatment, the biosolids showed a drastic reduction in *E*. *coli* and *Salmonella* content when looking at the enumeration of classic pathogens on growth media. Although the starting communities differed slightly, the different sewage treatments did affect the communities differently. From the results of the classic enumeration methods, the anaerobic digestion method of Plant A appeared less efficient in reducing the pathogen load in biosolids as compared to the dewatering/pelletization method of Plant C. Plant A exceeded the Canadian Food Inspection Agency (CFIA) criteria for *Salmonella* levels which must be non-detectable (at 3 out of 3 sampling dates, post-treatment biosolids had detectable *Salmonella*) and the level of fecal coliforms, which must not exceed 1000 MPN/g of the total dry weight (at 3 out of 3 sampling dates, treated biosolids exceeded that value) [18]. By contrast, treated biosolids from Plant C met the CFIA indicator standards at all sampling dates.

The dewatering/pelletization treatment also resulted in a larger and more consistent decrease in extractable DNA than the anaerobic digestion treatment. However, molecular methods highlighted stronger shifts in community composition following anaerobic digestion as compared to dewatering/pelletization. These larger microbial community composition shifts at Plant A as compared to Plant C might have been caused by the differences in the initial communities between the two plants, but, alternatively, it might have been a direct cause of the anaerobic environment itself, as many of the dominant groups of bacteria after anaerobic digestion were from known anaerobic microorganisms within the *Firmicutes* and *Bacteroidetes* phyla.

Molecular methods identified DNA originating from many genera, containing pathogenic species and virulence genes, that increased in their relative abundance following biosolid treatments. For instance, sewage treatment at Plant A increased the relative abundance of *Clostridium DNA* in biosolids whereas *Clostridium* DNA was also detected before and after the dewatering/pelletization treatment at Plant C. Even though many species of *Clostridium* are non-pathogenic, some are the causative agents of diseases in humans, including botulism, tetanus and enterocolitis. As such, their persistence in biosolids could be a cause for concern if these organisms were present in a viable state and at concentrations that could cause disease. Consistent with the work presented here, DNA related to *Clostridium* was detectable by 16S rRNA gene pyrosequencing after biosolids treatment [30]. Clostridia are obligate anaerobes which produce endospores, therefore their increase following anaerobic digestion at Plant A is not surprising since endospores can resist this type of treatment. The detection of DNA from potential pathogenic organisms other than *Salmonella* and fecal coliforms in post-treated samples emphasize the need for further research in order to determine the validity and applicability of indicator organisms currently used for regulatory purposes. The shotgun metagenomic approach also allowed the detection of numerous virulence-related genes in the processed biosolids of Plant A, including mobile genetic elements and antibiotic resistance genes, as previously reported in treated biosolids [31].

### Emerging methods to detect pathogens in biosolids

Culture-based methods, although the simplest and most inexpensive way to detect live pathogens, cannot detect viable but non-culturable (VBNC) bacteria which can potentially reanimate in anaerobically digested biosolids. [32, 33]. In contrast, at the molecular level, qPCR can detect non-culturable bacteria and could be used to detect the presence of a specific pathogen’s gene in biosolids [34, 35]. However, the wide range of possible pathogenic targets again renders such a method rather cumbersome. In our study, qPCR was not able to detect *Salmonella* in many samples, while it was detected by culture-based methods, *cpn60* pyrosequencing and metagenomic sequencing. This was surprising since the presence of VBNC bacteria, dead cells and naked DNA should have made the qPCR method detect more of this pathogen [34, 36]. Previous studies had already reported a lower sensitivity for qPCR than for culture methods [37], mainly related to the starting sample size. Further studies using spiked samples would be necessary to identify which method was the most sensitive, specific and reproducible.

In this study, we compared amplicon sequencing of *cpn60* and two regions of the 16S rRNA gene [38] and shotgun metagenomics [39–42] as potential alternatives to culture-based approaches for the detection of genetic material from pathogens in biosolids. The region of the 16S rRNA gene that was sequenced did not have a strong influence on the community composition at the genus and phylum levels. The main advantage of shotgun metagenomics, apart from avoiding any possible amplification bias, is that detection is not limited to the targeted organisms. Culture methods and qPCR can only detect the DNA of organisms that are targeted [34, 35, 43], while metagenomics and amplicon sequencing can detect DNA from all organisms present in a sample (including eukaryotes and viruses). For instance viruses, which can be an indicator for biosolid treatment efficiency, can represent up to 10–14% of total sequences in metagenomic datasets [44] but were not detected using other methods. Another striking example is that, even with a general decrease of *E*. *coli*, several other potential human pathogen DNAs like those from *Clostridium* increased significantly as previously reported using 16S rRNA pyrosequencing [30]. However, standard protocols for amplicon and metagenomic sequencing are only semi-quantitative (relative abundance), reducing the utility of the data. Using internal spiked standards like previously done in metatranscriptomics [45, 46] could help solve this issue. In the present study, if pathogen monitoring methods were constrained only for *E*. *coli* detection using culture-based methods, our data would have falsely indicated that the biosolids treatments were highly efficient in reducing pathogen loads in treated biosolids. Another major advantage of shotgun metagenomic sequencing is the ability to detect the whole complement of functional genes in an environmental sample; however, it is closely linked to the quality of the databases used. As the number of annotated species increases and are deposited into relevant databases, the similarity search against annotated genes will be more successful. It is especially true in the case of biosolids metagenomics, as human pathogens are one of the most sequenced and accurately annotated type of microorganism in current databases. Another critical aspect of current metagenomic studies is the read length used which 1) often precludes the definition of the context surrounding the genes detected, 2) cannot link the reads to an organism or a function with a very high level of certainty. The increased throughput of long-read sequencers could probably solve some of these issues in the near future.

Our data suggests that shotgun metagenomic sequencing could be an excellent supplementary method to culture-based methods for pathogen detection, which is the primary method used by the Canadian Food Inspection Agency to detect pathogens. Metagenomics has not only detected all the specific DNA of the organisms detected by other methods, but it had also detected scores of potential pathogens and virulence related genes that the other methods were unable to detect. However, this approach incurs significant costs and requires advanced analytical expertise. Until the cost of library preparation and sequencing decreases further and analyses become routine, our data shows that other powerful approaches like amplicon sequencing will work with the appropriate depth of sequencing. DNA-based methods, as used in this study, can indirectly determine the presence of pathogens through their DNA and virulence-associated factors, but cannot directly determine live pathogen counts. In future studies, it will be important to assess the presence of live pathogenic microbial cells, particularly viable but non-culturable organisms, by using more quantitative molecular approaches such as propidium monoazide (PMA) treatment [47, 48] in order to discriminate between live and dead cells.
